# Glycolysis and the significance of lactate in traumatic brain injury

**DOI:** 10.3389/fnins.2015.00112

**Published:** 2015-04-08

**Authors:** Keri L. H. Carpenter, Ibrahim Jalloh, Peter J. Hutchinson

**Affiliations:** ^1^Division of Neurosurgery, Department of Clinical Neurosciences, University of CambridgeCambridge, UK; ^2^Wolfson Brain Imaging Centre, Department of Clinical Neurosciences, University of CambridgeCambridge, UK

**Keywords:** traumatic brain injury (human), cerebral energy metabolism, glycolysis, lactate, pyruvate, glucose, microdialysis

## Abstract

In traumatic brain injury (TBI) patients, elevation of the brain extracellular lactate concentration and the lactate/pyruvate ratio are well-recognized, and are associated statistically with unfavorable clinical outcome. Brain extracellular lactate was conventionally regarded as a waste product of glucose, when glucose is metabolized via glycolysis (Embden-Meyerhof-Parnas pathway) to pyruvate, followed by conversion to lactate by the action of lactate dehydrogenase, and export of lactate into the extracellular fluid. In TBI, glycolytic lactate is ascribed to hypoxia or mitochondrial dysfunction, although the precise nature of the latter is incompletely understood. Seemingly in contrast to lactate's association with unfavorable outcome is a growing body of evidence that lactate can be beneficial. The idea that the brain can utilize lactate by feeding into the tricarboxylic acid (TCA) cycle of neurons, first published two decades ago, has become known as the astrocyte-neuron lactate shuttle hypothesis. Direct evidence of brain utilization of lactate was first obtained 5 years ago in a cerebral microdialysis study in TBI patients, where administration of ^13^C-labeled lactate via the microdialysis catheter and simultaneous collection of the emerging microdialysates, with ^13^C NMR analysis, revealed ^13^C labeling in glutamine consistent with lactate utilization via the TCA cycle. This suggests that where neurons are too damaged to utilize the lactate produced from glucose by astrocytes, i.e., uncoupling of neuronal and glial metabolism, high extracellular levels of lactate would accumulate, explaining the association between high lactate and poor outcome. Recently, an intravenous exogenous lactate supplementation study in TBI patients revealed evidence for a beneficial effect judged by surrogate endpoints. Here we review the current state of knowledge about glycolysis and lactate in TBI, how it can be measured in patients, and whether it can be modulated to achieve better clinical outcome.

## Introduction

Cerebral metabolism following injury appears to differ from that in normal brain, although the full extent and nature of these changes are poorly understood, especially in man.

Traumatic brain injury (TBI) results from the action of external mechanical forces that cause macroscopic tissue damage at the time of injury and initiate cellular processes that evolve over the hours and days that follow. It is a heterogeneous disorder that includes a range of macroscopic tissue pathologies including hematomas, contusions, and edema. Often these lesions are sufficiently large to require urgent surgical evacuation to prevent fatal compression of vital brain structures. Occasionally, even in the presence of severe TBI, defined clinically as patients presenting in coma, there is little in the way of macroscopic damage to the brain evident on CT scans. This illustrates the impact that pathophysiology at the microscopic level has on cellular and neurological function. These processes are multifactorial and include disturbed ion hemostasis (Unterberg et al., 2004), excitotoxicity (Katayama et al., 1990; Kawamata et al., 1992), cell wall and mitochondrial disruption (Xiong et al., 1997; Lewen et al., 2001), inflammation, and derangements in oxidative energy metabolism (Jalloh et al., 2014).

Patients with severe TBI frequently have injuries to other parts of the body such as the lungs or limb bones and are typically managed in an intensive care setting (Chesnut et al., 1993; Stocchetti et al., 1996). They are medically complex patients with a myriad of attendant issues that need to be considered, for example, nutrition, respiratory care, and deep-vein thrombosis prophylaxis, in addition to complications associated with TBI such as seizures, hydrocephalus, and endocrine dysfunction. Overall, TBI is one of the leading causes of mortality and morbidity in young adults. The most recent large observational studies report unfavorable outcomes from severe TBI ranging between 54 and 66% (Roozenbeek et al., 2013).

The best-known metabolic characteristics of injured brain are a high lactate concentration and a high lactate/pyruvate (L/P) ratio, in the brain extracellular fluid. An L/P ratio greater than 25 is interpreted as arising from high glycolytic activity indicative of hypoxia or mitochondrial dysfunction (Timofeev et al., 2011a), although the precise nature of the latter is incompletely understood. A recent study in 233 TBI patients demonstrated that L/P ratio >25 predicted unfavorable clinical outcome in a multivariate analysis in addition to previously known predictors of outcome (Timofeev et al., 2011a). Compared with normal brain, the course of the glycolytic pathway from glucose to lactate differs after injury, with in some cases an increase in the proportion of lactate produced via diversion of glucose-6-phosphate through the pentose phosphate pathway (PPP), also termed hexose monophosphate shunt, compared with direct glycolysis that even so remains the major route (Bartnik et al., 2005, 2007; Dusick et al., 2007; Jalloh et al., 2013).

## Measurement of glycolysis and lactate

**Figure 1:**
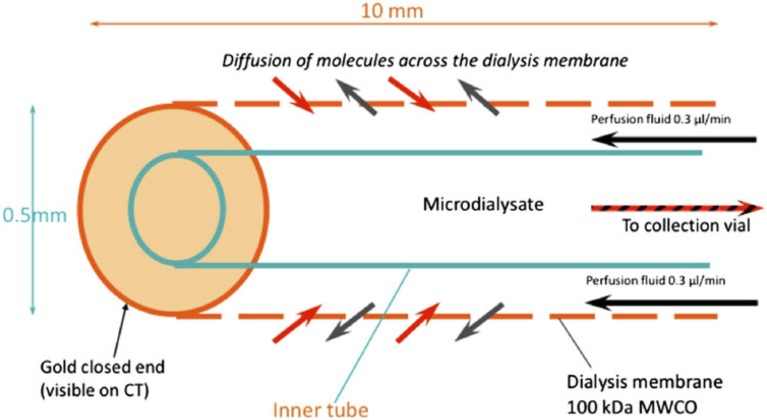
**Schematic of the microdialysis catheter tip**. Substances in the extracellular fluid outside the catheter tip are able to diffuse across the microdialysis membrane to be collected for analysis. Abbreviation: MWCO, nominal molecular weight cut-off of the microdialysis membrane. Originally published by Shannon et al. (2013) in J Pharmacokinet Pharmacodyn 40: 343–458 under a Creative Commons Attribution Licence.

Glycolysis is a multi-stage pathway with many facets, and there are various biochemical approaches to measuring glycolysis. Microdialysis is a tool for monitoring cerebral metabolism. Monitoring of severe TBI patients in neurocritical care may include intracranial pressure, brain tissue oxygen and extracellular chemistry using microdialysis. The latter possesses a semi-permeable membrane that is continuously perfused with fluid, allowing molecules to diffuse across the membrane, to and from the brain's extracellular space. Clinically, the catheter is perfused with a physiological salt solution and the returning fluid (microdialysate) is analyzed at the bedside utilizing automated enzymatic colorimetric assays to measure endogenous glucose, lactate, pyruvate, glutamate, and glycerol. In this way microdialysis has been used to monitor glucose delivery to the brain and, using L/P ratio, as a marker of the balance between “aerobic” (referring to TCA cycle) and “anaerobic” metabolism (referring to glycolysis culminating in lactate). A schematic diagram of the microdialysis catheter is shown in Figure 1.

In the laboratory, a classic method of measuring “true glycolysis” (the Embden-Meyerhof-Parnas pathway, also termed the Embden-Meyerhof pathway), is to measure radioactive water produced from [5-^3^H]-glucose, used originally in *ex vivo* organ (heart) perfusion (Neely et al., 1972) and more recently *in vitro* in cell cultures (e.g., De Bock et al., 2013). A limitation of this assay is that it does not measure conversion of glucose all the way through to pyruvate, and since it uses long-lived radioactivity in the form of tritium it is unsuitable for use in humans. A possible alternative would be to adapt the method by using the stable isotope deuterium in place of radioactive tritium and quantify the deuterated water by mass spectrometry. However, this would require a specialized type of mass spectrometry that is not widely available.

Besides the above, there are various other techniques and instrumentation for measuring aspects of glycolysis in the laboratory, reviewed recently (TeSlaa and Teitell, 2014). These are performed under controlled conditions, often in cell cultures with specific inhibitors to tie down particular components of biochemistry, although general principles such as measurement *in vitro* of extracellular levels of lactate, glucose and concentration of oxygen are also shared with neurocritical care monitoring *in vivo*. We have already mentioned microdialysis (above) for measuring brain extracellular glucose, lactate etc., and brain tissue oxygen (PbtO_2_) can be measured alongside using an intracranial oxygen sensor (Timofeev et al., 2011b; Shannon et al., 2013; Jalloh et al., 2015). Extracellular acidification measurement, employed *in vitro*, has also been performed in patients using the Neurotrend sensor to measure brain extracellular pH, although no further measurements are possible as the manufacturer (Diametrics, Buckinghamshire, UK) has discontinued this sensor and no alternative clinically approved intracranial pH sensor currently exists (Timofeev et al., 2013). Another glycolysis-related measurement is uptake of ^18^F-deoxyglucose (FDG) by cells. FDG is a glucose analog that becomes phosphorylated inside the cells and retained without further metabolism. FDG is more often employed in patients (as opposed to cell cultures), with clinical imaging by positron emission tomography (PET) as exemplified below.

**Figure 2:**
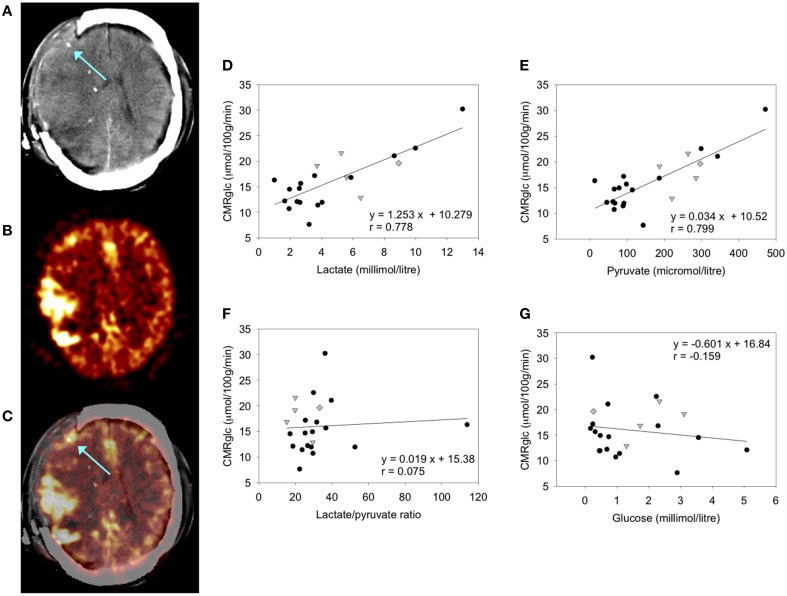
**FDG-PET measurement of CMRglc and its relationship to brain microdialysate composition: (A–C) FDG-PET CMRglc map demonstrating relatively high FDG uptake at sites of injury, in contrast to less injured areas of the brain. (A)** Computed tomography (CT) scan showing gold tip of microdialysis catheter (indicated by arrow). **(B)** Co-registered FDG-PET CMRglc map showing high FDG uptake at sites of injury. **(C)** Overlay of CT and co-registered CMRglc map, showing microdialysis catheter tip location (arrow). **(D–F)** Graphs illustrating relationships by linear regression (for 22 ROIs in 17 TBI patients) between FDG-PET derived CMRglc and the microdialysis parameters measured during the scan **(D)** lactate, **(E)** pyruvate, **(F)** lactate/pyruvate (L/P) ratio, and **(G)** glucose. For the linear regressions in **(D–G)**, corresponding values of *p* (ANOVA) are <0.0001, <0.0001, 0.74, and 0.48, respectively. Data-points from catheters at craniotomy sites (four patients) are differentiated by gray triangles. Data-points from a second FDG-PET scan (one patient) are differentiated by gray diamonds. All other data-points are depicted as black circles (catheters inserted via cranial access device). Linear regressions presented on the graphs are for the entire (combined black plus gray symbols) dataset consisting of all 22 ROIs. Originally published by Hutchinson et al. (2009) in Acta Neurochir (Wien) 151: 51–61, and reproduced with kind permission of Springer Science+Business Media.

Various studies of FDG-PET have been carried out in TBI patients, for example (Vespa et al., 2005; Hutchinson et al., 2009). Established computation methods allow cerebral metabolic rate of glucose (CMRglc, expressed in units of μmol/100 g tissue/min) to be determined for the desired regions of interest (ROIs), e.g., a 2 cm ROI around the microdialysis catheter tip (Hutchinson et al., 2009). While FDG-PET cannot distinguish glycolysis from TCA cycle metabolism, the use of microdialysis provides information on extracellular lactate, pyruvate and glucose concentrations and allows inferences to be made. A combined FDG-PET and microdialysis study showed significant positive correlations of CMRglc with lactate and pyruvate concentrations, no relationship between CMRglc and L/P ratio, and a weak inverse trend for CMRglc with glucose concentrations in the microdialysates (Figure 2) (Hutchinson et al., 2009). The study concluded (with the caveat of being a small study in 17 patients) that in TBI brain, an increase in glucose metabolism leads to increases in both lactate and pyruvate, as opposed to a shift toward “anaerobic” metabolism (Hutchinson et al., 2009). Only 2 of the 17 patients in this study showed very high L/P ratio exceeding 40 (Hutchinson et al., 2009), a state known as metabolic crisis. In a different study of microdialysis combined with PET (FDG and triple oxygen), a higher proportion (7 out of 19) of patients showed metabolic crisis (L/P ratio >40) though only a single patient showed regional ischemia (Vespa et al., 2005). Unlike (Hutchinson et al., 2009), there was no correlation between microdialysis parameters and regional CMRglc (Vespa et al., 2005). The apparent disparity between the two studies' results was suggested to have stemmed at least partly from the different proportions of metabolic crisis patients (Hutchinson et al., 2009).

**Figure 3:**
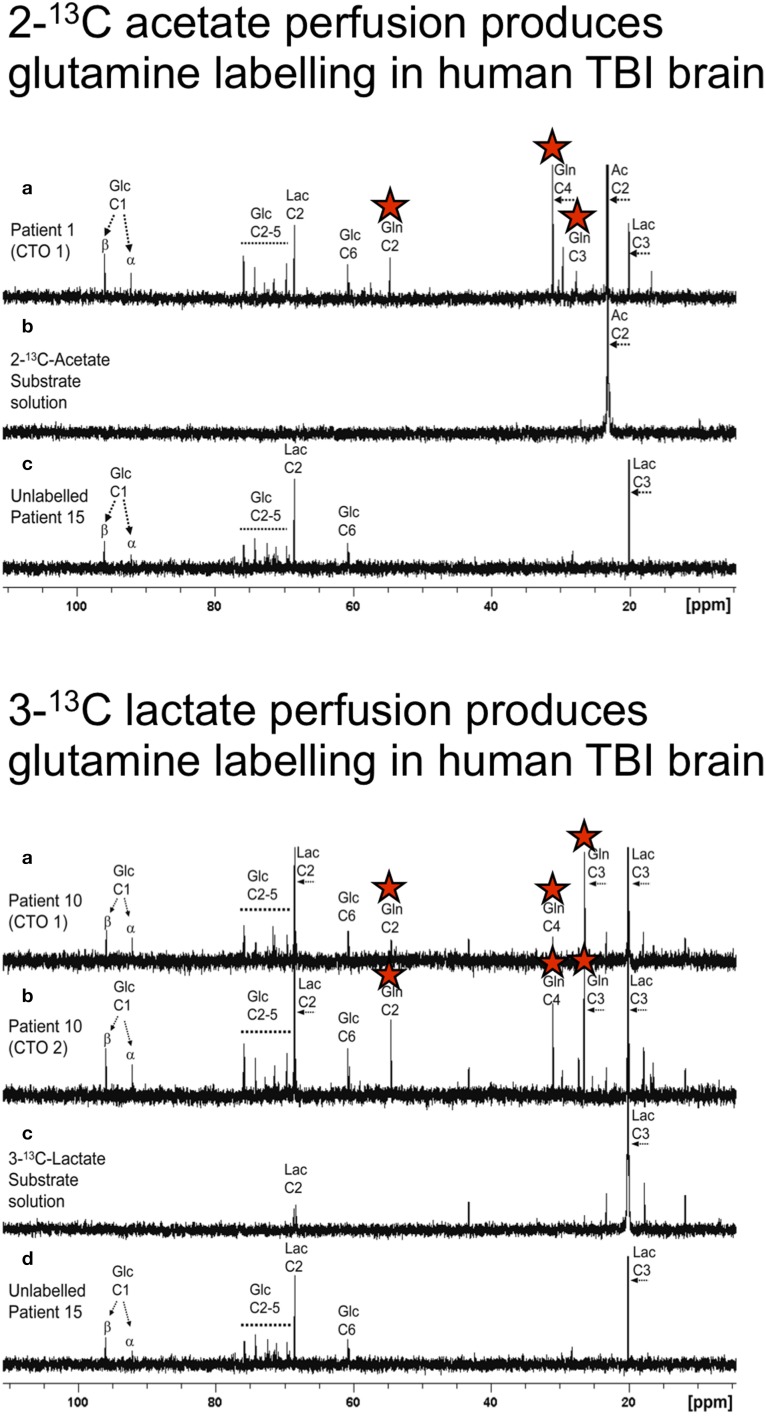
**Upper panel: (a) Example of ^13^C NMR spectrum of brain microdialysate from a TBI patient, who received perfusion with 2-^13^C acetate (4 mM) by a microdialysis catheter via a craniotomy (CTO); red stars indicate ^13^C signals for glutamine C4, C3, and C2 indicating metabolism via TCA cycle. (b)** ^13^C NMR spectrum of the 2-^13^C acetate substrate solution prior to perfusing. **(c)** ^13^C NMR spectrum of brain microdialysate from an unlabeled patient whose microdialysis catheter was perfused with plain perfusion fluid without labeled substrate. Lower panel: **(a,b)** Examples of ^13^C NMR spectra of brain microdialysates from a TBI patient, who received perfusion with 3-^13^C lactate (4 mM) by microdialysis catheters via a craniotomy (CTO); red stars indicate ^13^C signals for glutamine C4, C3, and C2 indicating metabolism via TCA cycle. **(c)** ^13^C NMR spectrum of the 3-^13^C lactate substrate solution prior to perfusing. **(d)** ^13^C NMR spectrum of brain microdialysate from an unlabeled patient [as in Upper panel **(c)**]. Originally published by Gallagher et al. (2009) in Brain 132: 2839–2849, and reproduced with permission of Oxford Journals.

As diffusion across the microdialysis membrane is bi-directional, microdialysis can also be used to deliver molecules (“retrodialysis” e.g., ^13^C-labeled substrates), thereby micro-dosing a region of interest around the catheter tip, whilst simultaneously collecting the products in the emerging microdialysate, for subsequent NMR analysis. In this way, we have infused 1,2-^13^C_2_ glucose into TBI patients' brains via the microdialysis catheter to compare production of glycolytic 2,3-^13^C_2_ lactate vs. PPP-derived 3-^13^C lactate (Jalloh et al., 2015). This study was carried out with brain tissue oxygen (PbtO_2_) measured simultaneously in the vicinity of the microdialysis catheter, shedding light on the relationship of local oxygen concentration to glycolytic- and PPP- generated lactate. Also, the ^13^C-labeling enabled newly synthesized lactate to be distinguished from “old” lactate, a differentiation otherwise impossible without labeling. The findings of the study (Jalloh et al., 2015) are discussed in the section entitled “*Origins of lactate in brain*,” below. We have also shown that infusion of 3-^13^C lactate or 2-^13^C acetate into the brains of TBI patients via the microdialysis catheter produced ^13^C signals for glutamine C4, C3, and C2 in the emerging microdialysates (analyzed by NMR), indicating TCA cycle operation followed by conversion of glutamate to glutamine (see Figure 3 and section entitled “*Brain utilization of lactate,”* below) (Gallagher et al., 2009). Microdialysis can thus be used to manipulate the immediate microenvironment around the catheter by adding chosen metabolic substrates that enter the relevant biochemical pathways at different stages allowing specific stages of the process to be investigated.

Brain TCA cycle flux in humans has been measured employing *in vivo* magnetic resonance spectroscopy (MRS) using ^13^C-labeled glucose (typically 1-^13^C glucose or 1,6-^13^C_2_ glucose) (Rothman et al., 2011). The TCA cycle flux is calculated by kinetic modeling of ^13^C labeling in glutamine and glutamate in brain measured by MRS, during ^13^C labeled glucose intravenous infusion. The technique has been mostly applied to healthy volunteers. A few *in vivo* MRS studies of ^13^C-labeling have been carried out in patients with various pathologies, but the technique has not yet been applied to the severe TBI field. Only a few centers worldwide have neurocritical care units adjacent to MRI scanners equipped for MRS and the expertise to support fully ventilated severe TBI patients through the procedure, and moreover, ^13^C measurement of TCA cycle is itself a highly specialized area within the *in vivo* MRS field. *In vivo* brain MRS studies of ^13^C labeling are extendable to other intravenously administered substrates; for example ^13^C acetate has been used (Lebon et al., 2002), and *in vivo* MRS has been used to examine ^13^C lactate utilization in the TCA cycle, by measuring ^13^C labeling kinetics in glutamate and glutamine in healthy volunteers (Boumezbeur et al., 2010). Reviewing the latter study, Rothman et al. (2011) commented as follows. “Relative consumption of plasma lactate between neurons and astrocytes is similar to that of glucose (Boumezbeur et al., 2010). The calculation of the lactate metabolic capacity is in good agreement with recent AV difference studies using isotopically labeled lactate as a tracer, further confirming the potential importance of plasma lactate as a substrate for brain metabolism (van Hall et al., 2009).”

**Figure 4:**
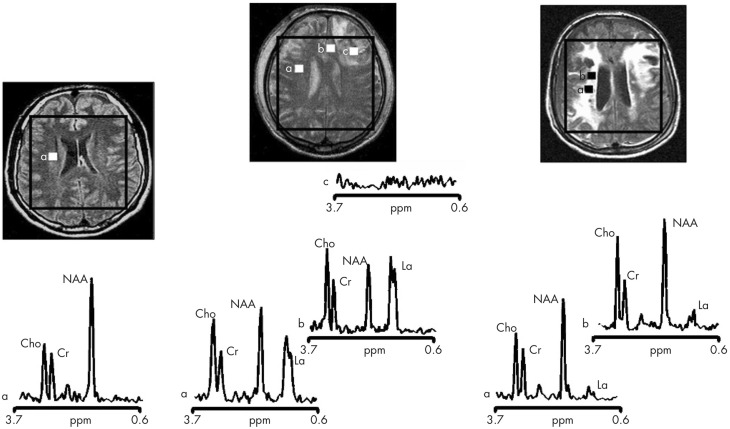
**Proton magnetic resonance spectra and conventional magnetic resonance images showing the volume of interest for spectroscopic imaging of a normal control (left panel), Patient 1 (central panel), and Patient 8 (right panel) with traumatic brain injury (TBI)**. On conventional MRI, Patient 1 shows a focal hematoma in the frontal left hemisphere and patient 8 shows diffuse MRI abnormalities. Spectra show decreases of N-acetylaspartate (NAA) and increases of choline (Cho) and lactate (La) in patients with TBI (a and b in central and right panels) with respect to the normal control (a in left panel). The spectra of Patient 1 (central panel) show more pronounced metabolic abnormalities than those of Patient 8 (right panel), despite the fact that Patient 8 showed markedly more abnormalities on conventional MRI. In the spectra of Patient 1 (central panel), metabolic abnormalities are clearly evident in the normal appearing brain. Finally, in Patient 1, voxels inside the focal hematoma (c in central panel) were excluded to avoid the artifacts that could be derived by the cerebral haemorrhagic contusion. Cr, creatine. Reproduced from J Neurol Neurosurg Psychiatry, Marino et al. 78: 501–507 (2007) with permission from BMJ Publishing Group Ltd.

More readily achievable than ^13^C *in vivo* MRS measurements of TCA cycle flux are ^1^H *in vivo* MRS measurements of endogenous molecules. ^1^H *in vivo* MRS in TBI clinically in patients (Figure 4) (Marino et al., 2007) have shown that the most abundant signal is N-acetylaspartate (NAA), a mitochondrial marker. As absolute quantification is difficult, NAA is often expressed as a ratio to creatine, or sometimes to choline (Marino et al., 2007; Maddock and Buonocore, 2012). While lactate is abundant extracellularly (at millimol/L concentrations readily measurable on microdialysis), it is, in contrast, much less evident on ^1^H *in vivo* MRS of normal or TBI brain. Moreover, depending on the choice of echo time for MRS, these small lactate signals can virtually disappear or appear inverted. Lactate elevation can be seen on ^1^H *in vivo* MRS in pathological states, e.g., in tumors. In TBI brain, lactate elevation can be seen on ^1^H MRS in some but not all instances (Marino et al., 2007).

Another *in vivo* modality is ^31^P MRS, which can inform on ATP in tissues, including brain, by measuring the phosphocreatine/ATP ratio and inorganic phosphate/ATP ratio (Garnett et al., 2001) or by ^31^P magnetization-transfer methods (Befroy et al., 2012). ^31^P has a natural abundance of 100% of all phosphorus atoms. While ATP is produced at a much higher yield (per molecule of glucose) by the combination of glycolysis plus mitochondrial metabolism than by glycolysis alone, the latter is thought to represent a quick source of ATP under stress-response conditions such as TBI. A combination of modalities is needed in order to differentiate between glycolysis and mitochondrial metabolism as sources of ATP in brain.

## Origins of lactate in brain

The majority of lactate in the brain is regarded as “glycolytic,” originating from glucose metabolism *in situ*, by the Embden-Meyerhof pathway, to pyruvate, followed by conversion of pyruvate to lactate by the action of lactate dehydrogenase (LDH). Other routes to brain lactate, including synthesis via the PPP and uptake of lactate from the circulation, are discussed below.

There is some disparity in nomenclature in the brain metabolism literature, which undoubtedly adds confusion for any non-biochemist readers, or even for biochemists unfamiliar with the brain injury field. In the brain injury literature, glycolysis culminating in lactate is often termed “anaerobic metabolism,” though often without supporting evidence regarding the oxygen status in the tissue concerned. In old studies brain injury was often associated with hypoxia/ischemia (real or assumed), although more recently advances in neurocritical care mean that overt hypoxia/ischemia is usually avoided. Even so, microvascular ischemia appears to exist in some cases (Newcombe et al., 2013), as do episodes of hypoxia (Timofeev et al., 2011b). We regard hypoxia as PbtO_2_ <20 mmHg, with severe hypoxia as PbtO_2_ <10 mmHg.

High brain lactate concentrations may result from mitochondrial dysfunction forcing cells to rely on glycolysis (Embden-Meyerhof pathway) to generate ATP. Without functional mitochondrial electron transport chains (ETC) and shuttles, the conversion (mediated by LDH) of pyruvate to lactate is necessary to recycle NADH to NAD^+^ to enable glycolysis to continue. Hypoxia prevents mitochondria from functioning properly. Since molecular oxygen (O_2_) is the terminal electron acceptor of the ETC, adequate O_2_ presence is vital for mitochondrial function. However, mitochondria may become dysfunctional even in the presence of O_2_, for instance if any of the ETC components are damaged, or if the TCA cycle (which feeds the ETC) is compromised, or if the mitochondrial membranes become leaky. The concentration of lactate in the brain depends not only on lactate production but also lactate consumption. We have previously commented, “Low extracellular lactate levels, associated with better outcomes (Timofeev et al., 2011a), might be because astrocytic glycolysis-derived lactate is being efficiently taken up by neurons and utilized via the TCA cycle (Gallagher et al., 2009). Conversely, high extracellular lactate may result if neurons are too damaged to efficiently utilize the lactate being produced by astrocytes, i.e., uncoupling of neuronal and glial metabolism” (Carpenter et al., 2014).

In classic biochemical terms, “glycolysis” is the Embden-Meyerhof pathway from glucose to pyruvate, whereas the pyruvate to lactate step is a subsequent conversion that is not part of glycolysis proper. Pyruvate does not necessarily produce lactate, as pyruvate can be converted into acetyl CoA and thence enter the TCA cycle. Also, lactate can be converted to pyruvate, and thence to acetyl CoA and the TCA cycle (see Section entitled “*Utilization of lactate*,” below). The description “glycolytic” is often loosely applied to cells or tissues that produce abundant lactate, e.g., injured regions of brain or tumors, and these cells/tissues also may have apparently elevated PPP as well as high glycolysis. In tumors, and especially in cancer cell lines *in vitro*, it has long been recognized that the high glycolysis often occurs despite a ready supply of oxygen, a phenomenon known as the Warburg effect (Warburg, 1956), often termed “aerobic glycolysis” in the tumor literature (Bensinger and Christofk, 2012). In a study of 24 TBI patients, Sala et al. (2013) judged that elevations in brain extracellular lactate were predominantly “glycolytic,” with “hypoxic” lactate elevation in a minority. These authors stated their criteria for “hypoxic lactate” as microdialysate lactate >4 mmol/L with PbtO_2_ <20 mmHg, and “glycolytic lactate” as microdialysate lactate >4 mmol/L with microdialysate pyruvate >119 mmol/L. It is relevant to comment that the actual origins of the lactate were not determined in this study. Therefore, the brain extracellular lactate detected may have included a minor portion arising via the PPP rather than direct glycolysis, and moreover some of the lactate may have entered the brain from the circulation; see below for further information. Results from CT perfusion (with iohexol contrast agent) available for a subset of (16 out of 24) patients in the study by Sala et al. suggested that “glycolytic” lactate was associated with hyperaemic brain perfusion, and “hypoxic” lactate with “diffuse oligaemia” (Sala et al., 2013).

Relevant to the balance between lactate and pyruvate is the nature of the LDH enzyme. Two distinct subunits are combined to form the five tetrameric isoenzymes of LDH. Subunit LDH1 (“heart type,” found in neurons) preferentially drives lactate to pyruvate, while subunit LDH5 (“muscle type”) is present in “glycolytic tissues” and in both neurons and astrocytes, and preferentially drives pyruvate to lactate (Bittar et al., 1996).

The PPP is a complex detour starting from glucose-6-phosphate (hence its alternative name “hexose monophosphate shunt”) bypassing some of the steps of glycolysis in the metabolism of glucose, and its key features have been summarized as follows by Jalloh et al. (2015). “The key enzyme for the PPP, glucose-6-phosphate dehydrogenase, which is responsible for the rate-limiting step, is present in most tissues and cell types, and is regarded as a “housekeeping” enzyme (Pandolfi et al., 1995; Riganti et al., 2012). The PPP does not consume or produce ATP and does not require molecular oxygen. In the early “oxidative phase” of the PPP, the first carbon of the glucose skeleton is lost as carbon dioxide, and nicotinamide adenine dinucleotide phosphate (NADP^+^) is converted to NADPH. The latter participates in reductive biosynthetic reactions, such as lipid synthesis and in producing the reduced form of glutathione and thioredoxin which are cofactors for glutathione peroxidase and peroxiredoxins respectively, both of which scavenge hydroperoxides, thereby combatting oxidative stress. Among the many intermediates of the later “non-oxidative” phase of the PPP is ribose 5-phosphate, used for nucleic acid synthesis. PPP activity after TBI has been suggested to play a protective role, promoting synthesis of nucleic acids and fatty acids for tissue repair and combatting oxidative stress (Ben-Yoseph et al., 1996; Bartnik et al., 2005).”

**Figure 5:**
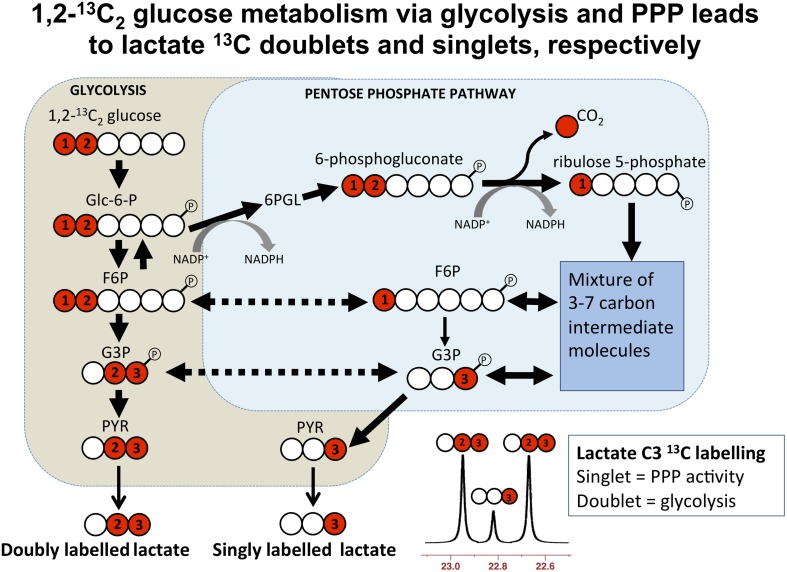
**Simplified schematic of steps in glycolysis and the pentose phosphate pathway (PPP), showing ^13^C labeling patterns resulting from 1,2-^13^C_2_ glucose substrate**. Red fills indicate ^13^C atoms. Abbreviations: Glc-6-P, glucose-6-phosphate; 6PGL, 6-phosphogluconolactone; F6P, fructose-6-phosphate; G3P, glyceraldehyde-3-phosphate; PYR, pyruvate. Originally published by Carpenter et al. (2014) in Eur J Pharm Sci 57: 87–97 under a Creative Commons Attribution Licence.

**Figure 6:**
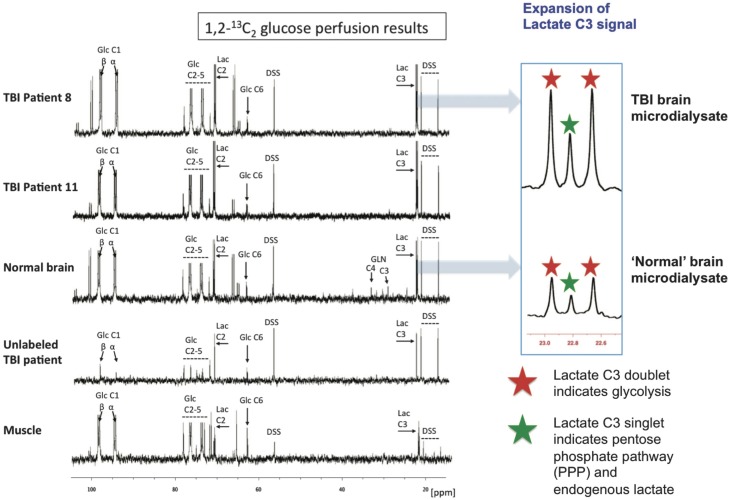
**Illustrative examples of ^13^C NMR spectra for microdialysates from patients who received perfusion with 1,2-^13^C_2_ glucose (4 mmol/L): TBI brain (uppermost two spectra, 24-h perfusion), “normal” brain (third spectrum, 24-h perfusion), and muscle (bottom spectrum, 8-h perfusion)**. An example of brain microdialysate from an unlabeled TBI patient with plain (unsupplemented) perfusion fluid is shown for comparison (fourth spectrum). Examples of expansion of the lactate C3 signal are shown for TBI brain and “normal” brain. Red stars indicate lactate C3 doublet (due to glycolytic 2,3-13C2 lactate) and green stars indicate C3 singlet (due to pentose phosphate pathway-derived 3-^13^C lactate plus endogenous lactate). Glucose (Glc), lactate (Lac), 4,4-dimethyl-4-silapentane-1-sulfonate sodium salt (DSS, the internal reference standard). Spectra were run from −20 to +250 p.p.m. The main reference DSS signal at 0 p.p.m. is not shown in the range illustrated. Originally published by Jalloh et al. (2015) in J Cereb Blood Flow Metab 35: 111–120, and reproduced with permission of Nature Publishing Group.

**Figure 7:**
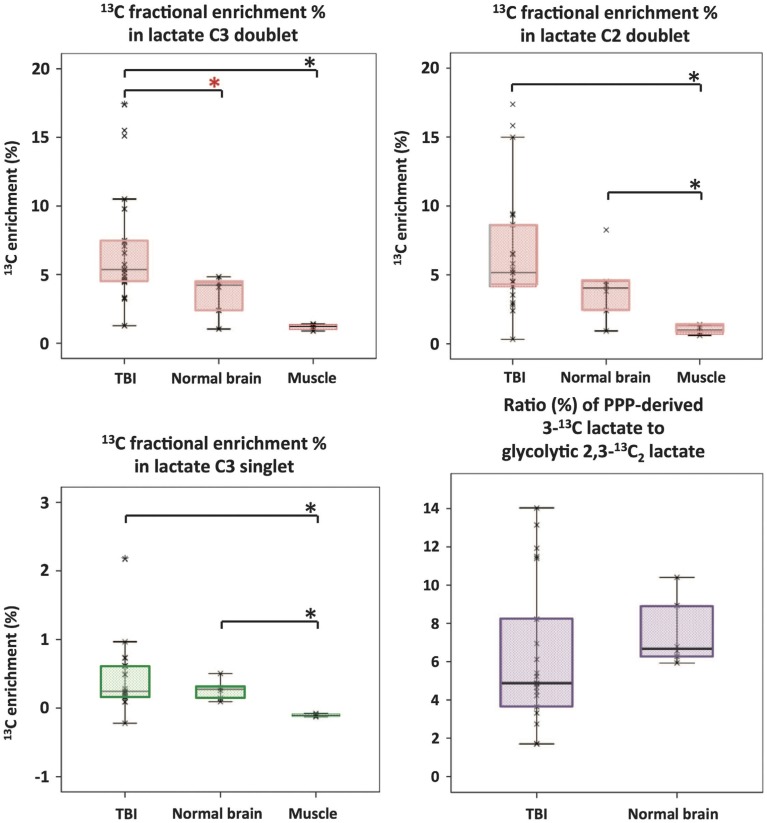
**Microdialysate NMR measurements of ^13^C labeling: results from perfusion for 24-h- (brain: TBI or “normal”) or 8-h perfusion (muscle) with 1,2-^13^C_2_ glucose (4 mmol/L)**. Red asterisks denote *P* < 0.01 for TBI vs. “normal” brain (Mann–Whitney); other comparisons asterisked in black denote *P* < 0.05. Individual data points are shown by × symbols. Number of patients: 15 TBI, six “normal” brain, and four muscle. Originally published by Jalloh et al. (2015) in J Cereb Blood Flow Metab 35: 111–120, and reproduced with permission of Nature Publishing Group.

**Figure 8:**
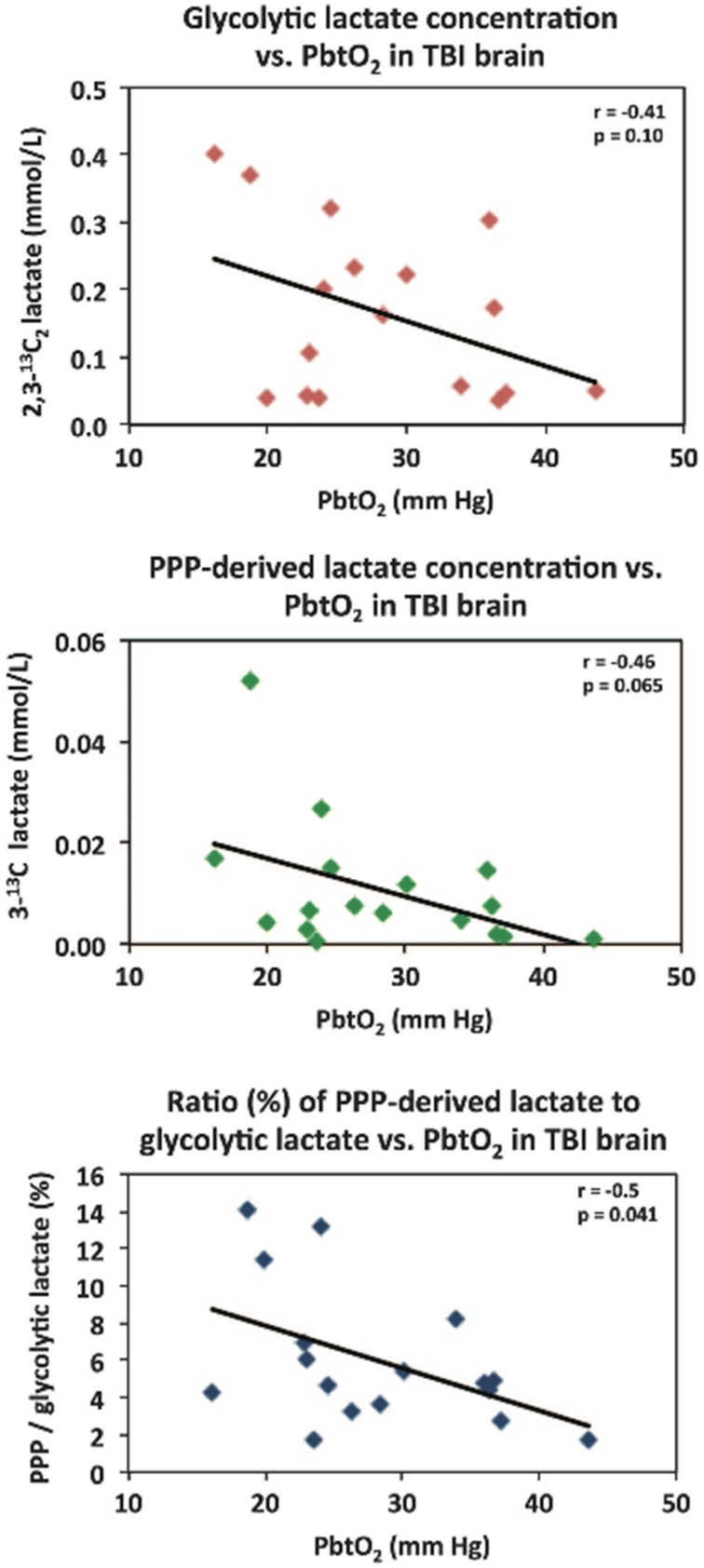
**Relationships in TBI brain for glycolytic lactate and pentose phosphate pathway (PPP)-derived lactate vs. PbtO_2_**. Concentrations of glycolytic 2,3-^13^C_2_ lactate (upper panel) and PPP-derived 3-^13^C lactate (middle panel) plotted vs. PbtO_2_. Lower panel: ratio (%) of PPP-derived 3-^13^C lactate to glycolytic 2,3-^13^C_2_ lactate, plotted vs. PbtO_2_. Each data point represents the results of NMR analysis of the combined contents of 24 × 1 h of microdialysate collection vials from one microdialysis catheter, plotted against the corresponding PbtO_2_ concentration expressed in mmHg, measured using a Licox oxygen probe placed alongside the microdialysis catheter in the brain. Lines are fitted by linear regression (statistics shown are Pearson's correlation coefficient r and analysis of variance *P*–value). Data are from 13 TBI patients. Four of these thirteen had a second period of monitoring, making 17 data points in total for each correlation. Originally published by Jalloh et al. (2015) in J Cereb Blood Flow Metab 35: 111–120, and reproduced with permission of Nature Publishing Group.

Microdialysis perfusion with 1,2-^13^C_2_ glucose, in TBI patients' brains, and, for comparison, in “normal” brain in non-TBI patients, with high-resolution ^13^C NMR analysis of the emerging microdialysates, has enabled comparison of lactate production by glycolysis (evidenced by 2,3-^13^C_2_ lactate), and the PPP (evidenced by 3-^13^C lactate) (Jalloh et al., 2015). Doubly labeled lactate (with ^13^C next to ^13^C) produces characteristic doublet signals on ^13^C NMR, distinct from singlets due to single ^13^C labeling (^13^C next to ^12^C). A schematic of the biosynthetic pathway labeling patterns is shown in Figure 5 (Carpenter et al., 2014; Jalloh et al., 2015), and illustrative examples of ^13^C NMR spectra in Figure 6 (Jalloh et al., 2015). The natural abundance of ^13^C is 1.1% of all carbon atoms, so the probability of two endogenous ^13^C atoms occurring next to each other naturally is 1.1 × 1.1% = 0.01%, thus the doublet signature, for doubly ^13^C labeled lactate, is essentially free from background. The singly ^13^C labeled lactate results were expressed after background-subtraction of the natural lactate's contribution to ^13^C NMR singlet signals. Glycolysis was always the major source of lactate and the PPP the minor source. The conclusions were as follows (Jalloh et al., 2015), and the graphs (Figures 7, 8) are from the same paper. “The minor pathway, PPP-derived lactate production, was statistically not significantly different in the TBI brain than in normal brain. However, several of the TBI individuals showed PPP-derived lactate elevation above the range observed in the normal brain (Figure 7). There was a shift in glucose metabolism from glycolysis to PPP with decreasing brain tissue oxygen (PbtO_2_) concentrations (Figure 8). The findings raise interesting questions about the roles of the PPP and glycolysis after TBI, and whether they can be manipulated to enhance the potentially reparative and antioxidant role of the PPP and achieve a better outcome for the patient. The ^13^C methodology developed here provides a means of distinguishing recently synthesized lactate and its biosynthetic origin, and at the same time measuring local oxygen tension alongside. ^13^C-labeled microdialysis with 1,2-^13^C_2_ glucose as substrate may thus find a methodological role in studies of hyperoxia or strategies to optimize perfusion and mitochondrial function. This is the first time that a comparison between glycolysis and the PPP has been carried out directly in the human brain.”

Studies in adult rats and brain slices suggested that the PPP increased after hypoxia (Domanska-Janik, 1988). Contrastingly, other researchers found that, in neonatal rats, the PPP decreased after hypoxia-ischemia, suggesting that inability to upregulate the PPP may render neonatal brain vulnerable to oxidative stress (Brekke et al., 2014).

Although the shared gateway into both the Embden-Meyerhof pathway and the PPP is via the action of hexokinase (HK) converting glucose to glucose-6-phosphate (G6P), there are several isoforms of that enzyme. HKI (dubbed “the brain HK”) seems to predestine G6P for subsequent processing via the Embden-Meyerhof pathway, while HKII (“the muscle HK”) has a more complex role and under some circumstances seems to predispose toward the PPP. There is evidence to suggest HK1 being located bound to mitochondria. The interaction of HKs with mitochondria is not static, but is regulated by factors such as glucose, G-6-P and kinases such as protein kinase B (PKB, also known as Akt) and glycogen synthase kinase 3 (GSK-3). Accordingly, HKII has been suggested to translocate between the cytosol, where it channels G6P into the PPP, and binding to mitochondria, where it predisposes G6P to “glycolysis and oxidative phosphorylation” (John et al., 2011). It has been suggested that shifts in expression (and/or activity) between HKI and HKII may be a contributing factor (e.g., in injury or disease) to changes in the balance between the Embden-Meyerhof pathway and the PPP (John et al., 2011).

Glucose-6-phosphate dehydrogenase (G6PD, also known as G6PDH), the enzyme responsible for the rate-limiting step of the PPP, is known to exhibit many gene mutations, some of which are manifest as various degrees of G6PD enzyme deficiency (Notaro et al., 2000; Cappellini and Fiorelli, 2008). The G6PD gene is on the X-chromosome, and has long been recognized in the context of the phenomenon of the mosaic of X-chromosome inactivation in females (Beutler et al., 1962), termed Lyon's Hypothesis or Law (Lyon, 1961; Gendrel and Heard, 2011; Harper, 2011). In female embryo early development, inactivation occurs randomly of one of the two X-chromosomes in each cell, then tissues and organs develop as mosaics consisting of clonal patches of cells that maintain their original inactivation state during subsequent cell divisions. G6PD can be inhibited by dehydroepiandrosterone (DHEA) (Rodriguez-Rodriguez et al., 2013). DHEA, a neurosteroid, is synthesized in brain, and also outside of brain (mainly by adrenal glands). DHEA is considered neuroprotective, and, in human brain tissue, there was a tendency for lower DHEA levels in subjects with Alzheimer's disease than in non-dementia similarly-aged controls (Schumacher et al., 2003). Endogenous DHEA levels are regarded as declining with age, and highest in males and lower in females, based on circulating levels which show individual variations and pathology-related variations (Samaras et al., 2013). DHEA in brain tissue is less well-understood. The above factors regarding G6PD and DHEA might thus contribute to variability in the PPP vs. glycolysis as sources of lactate.

Another source of lactate in the brain, besides production *in situ* from glucose via glycolysis (major pathway) and PPP (minor pathway) is uptake of lactate from the circulation (Ide et al., 2000; Overgaard et al., 2012). The brain shows periods of net uptake and net export of lactate. This has been studied by arteriovenous (AV) difference in TBI patients by Jalloh et al. (2013). Their conclusions were as follows (Jalloh et al., 2013). “Our findings suggest that the injured brain takes up lactate, which can be oxidatively metabolized (Gallagher et al., 2009). Lactate uptake occurs despite relatively high brain lactate levels after TBI suggesting up-regulation of MCT transporters. Glucose delivery to brain cells is maintained during periods of lactate uptake. Hence, lactate uptake may reflect an adaptive response to the increased energy demands and change in metabolic priorities of the injured brain. The injured brain's capacity to use endogenous lactate as an alternative fuel implies that exogenous lactate may be therapeutic in TBI patients. Accordingly, rats given intravenous lactate after fluid percussion injury performed better than those given saline (Rice et al., 2002; Holloway et al., 2007). Glycemic control of neurocritical care patients is necessary to avoid both hypo- and hyperglycaemia, although tight glycaemic control may be too restrictive for optimal cerebral metabolism and less rigid control may be preferable (Kramer et al., 2012). Lactate administration may have a role in supporting energy metabolism in this context. Ongoing and future clinical studies will elucidate whether lactate administration improves outcomes.”

A possible contribution to lactate in brain is via the TCA cycle (Cruz and Cerdan, 1999; Tyson et al., 2003; Sonnewald, 2014). The TCA cycle intermediates malate and oxaloacetate (OAA) can be converted to pyruvate by the action of malic enzyme; also OAA can be converted by the action of phosphoenolpyruvate carboxykinase (PEPCK) plus pyruvate kinase (PK) to pyruvate, then LDH can convert pyruvate to lactate. Anaplerosis (i.e., topping-up) of TCA cycle intermediates is coupled to cataplerosis (i.e., spin-out) from the TCA cycle, and Sonnewald has recently hypothesized, “cataplerosis in the brain is achieved by exporting the lactate generated from TCA cycle intermediates into the blood and perivascular area…. This shifts the generally accepted paradigm of lactate generation as simply derived from glycolysis to that of oxidation and might present an alternative explanation for aerobic glycolysis” (Sonnewald, 2014). Whether this is a significant route of lactate production in human TBI brain is not (to our knowledge) reported in the literature.

## Utilization of lactate in TBI

**Figure 9:**
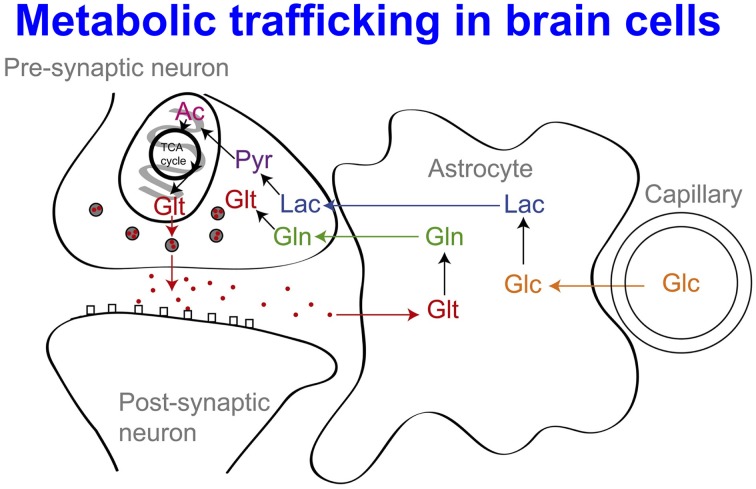
**Glucose (Glc) from the vasculature is metabolized to lactate (Lac) in astrocytes, exported into the extracellular fluid, taken up by neurons and processed [via pyruvate (Pyr) and acetate (Ac)] by the TCA cycle**. This spins off glutamate (Glt), which is released and then taken up by astrocytes, which convert it to glutamine (Gln), which is released into the extracellular fluid and taken up by neurons, which re-convert it to glutamate. For details of the relevant membrane transporters (see Chih and Roberts, 2003; Gallagher et al., 2009; Pellerin and Magistretti, 2012). Besides Lac production *in situ* from Glc, Lac may also be taken up from the circulation, (see Ide et al., 2000; Overgaard et al., 2012; Jalloh et al., 2013). For typical concentrations of the above species and PbtO_2_ (see Gallagher et al., 2009; Timofeev et al., 2011a,b). Originally published by Gallagher et al. (2009) in Brain 132: 2839–2849, and reproduced with permission of Oxford Journals.

While glucose is recognized as the primary energy substrate in most organs, recent evidence suggests that the situation in the brain is considerably more complex, particularly in relation to the main cell types. Traditionally it has been believed that both glia and neurons metabolize glucose as the preferred substrate via glycolysis to pyruvate, which is converted to acetyl CoA and enters the TCA cycle resulting in the generation of ATP by oxidative phosphorylation. Recent evidence suggests, however, that the neurons may utilize lactate (classically perceived as a waste product) as an energy substrate. Figure 9 outlines a scheme of metabolic trafficking between astrocytes and neurons (Gallagher et al., 2009). Glucose (Glc) from the vasculature is metabolized to lactate (Lac) in astrocytes, exported into the extracellular fluid, taken up by neurons and processed (via pyruvate and acetate) by the TCA cycle. This spins off glutamate (Glt), which is released and then taken up by astrocytes, which convert it to glutamine (Gln), which is released into the extracellular fluid and taken up by neurons, which re-convert it to glutamate (Gallagher et al., 2009). This theory, which has become known as the astrocyte-neuron lactate shuttle (ANLS) hypothesis was proposed by Pellerin and Magistretti (1994) as a result of *in vitro* studies and has been later supported by studies in animals, (e.g., Tyson et al., 2003). Moreover, our recent microdialysis studies using ^13^C-labeling have demonstrated that the injured human brain can metabolize lactate via the TCA cycle (Gallagher et al., 2009). We suggested that low extracellular lactate levels, with better outcomes, might be because astrocytic glycolysis-derived lactate is being efficiently taken up by neurons and utilized via the TCA cycle (Gallagher et al., 2009). Conversely, where neurons are too damaged to utilize the lactate produced from glucose by astrocytes, i.e., uncoupling of neuronal and glial metabolism, high extracellular levels of lactate would accumulate, with poor outcome (Carpenter et al., 2014).

Although the ANLS hypothesis is still somewhat controversial, in recent years the concept of lactate as a brain energy substrate has been extended even further by Schurr in the postulation that lactate (rather than pyruvate) may be the true substrate for mitochondrial respiration (Schurr, 2006). In Schurr's postulated scheme, the glycolytic route in the cytosol, from glucose to pyruvate then by LDH-mediated conversion to lactate, is followed by uptake of lactate by a monocarboxylate transporter (MCT) in the mitochondrial outer membrane, then by conversion of lactate to pyruvate (by LDH1?) in the mitochondrial inner membrane (Schurr, 2006). The rest of the biochemical pathway of mitochondrial respiration (pyruvate to acetate CoA, TCA cycle, ETC and ATP synthesis) then follows on.

More recent studies of the significance of lactate led Suzuki et al. to propose, “astrocyte-neuron lactate transport is essential for long term synaptic plasticity, long-term memory, and its underlying molecular and synaptic changes” (Suzuki et al., 2011). Lactate transport may thus have important implications for pathologies with cognitive deficits. Very recently, Galow et al. showed that gamma oscillations, required for complex neuronal processing, can be fuelled by energy-rich substrates, most effectively by high concentrations of glucose, but also, to a somewhat lower degree, by high concentrations of lactate or pyruvate (Galow et al., 2014).

A recent study by Bouzat et al. showed evidence for beneficial effects (judged by surrogate markers) resulting from intravenous lactate administration in 15 TBI patients (Bouzat et al., 2014). A mixed effects linear regression model was used to evaluate the results. The treatment caused increases in brain microdialysate lactate (coefficient 0.47 mmol/L, 95% confidence interval 0.31–0.63 mmol/L), pyruvate [13.1 (8.78–17.4) μmol/L], and glucose [0.1 (0.04–0.16) mmol/L; all *p* < 0.01]. Reductions in brain microdialysate glutamate [−0.95 (−1.94–0.06) mmol/L, *p* = 0.06] and ICP [−0.86 (−1.47–0.24) mmHg, *p* < 0.01] were also observed (Bouzat et al., 2014). The lactate solution administered was hypertonic, and it was not resolved how much of the apparent benefit on ICP was due to this property or due to the actual lactate itself.

An earlier study by Ichai et al. in TBI patients showed that hyperosmolar sodium lactate solution (504 mmol/L; 1100 mosm/L) given intravenously was more effective at lowering ICP than a mannitol solution with an equivalent osmotic load (1160 mosm/L) (Ichai et al., 2009). Also, compared with saline solution (0.9%), a 0.5 mol/L sodium lactate solution was more effective at reducing the occurrence of raised ICP episodes in TBI patients (Ichai et al., 2013). In a model of TBI–controlled cortical impact (CCI) in rats–infusion of lactate (100 mmol/L) showed significantly improved cerebral blood flow (CBF), significantly reduced lesion volume, and no significant difference in extracellular glutamate concentration, when compared to CCI with saline (0.9%) infusion control (Alessandri et al., 2012).

**Figure 10:**
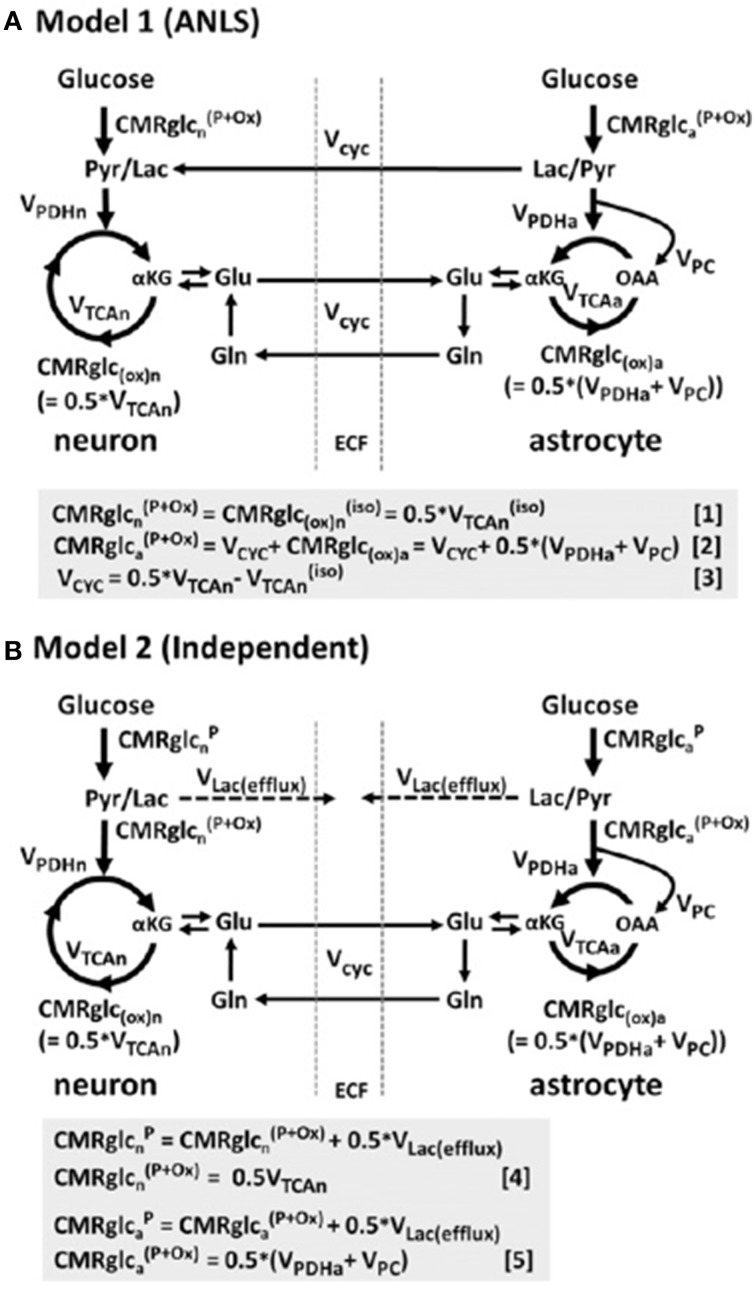
**Schematic depiction of two neuroenergetics models under consideration to account for the 1:1 flux relationship between increments in the rate of glutamate/glutamine cycling (V_cyc_) and the TCA cycle flux in neurons (V_TCAn_). (A)** ANLS-type model (Model 1). **(B)** Independent-type model (Model 2) in which neurons and astrocytes take up and oxidize glucose according to their respective energy needs. Phosphorylated glucose not oxidized within the cell may be effluxed as lactate, V_Lac(efflux)_, which is shown by dashed lines. The results of this study in rat brain suggested that neurons are capable of supporting a substantial fraction of their substrate requirements by direct uptake and phosphorylation of glucose. Originally published by Patel et al. (2014) in Proc Natl Acad Sci USA 111: 5385–5390, and reproduced with permission from Proc Natl Acad Sci USA.

Furthermore, such studies of intravenous lactate and the concept of the ANLS model have elicited controversy, and other models have been proposed (Dienel, 2014). Much of the evidence for the ANLS model is summarized in a review (Pellerin and Magistretti, 2012). A recent kinetic modeling study in rats (using non-radioactive ^19^F-FDG and 1,6-^13^C_2_ glucose) compared an ANLS model with an “independent” model in which neurons and astrocytes take up and oxidize glucose according to their respective energy needs (Patel et al., 2014) (Figure 10). The results did not support the ANLS astrocytic lactate production shuttling to neurons to provide a major neuronal fuel, but instead favored the “independent” model with neuronal glucose-derived pyruvate as the major oxidative fuel for activated neurons (Patel et al., 2014).

Compatible with the conclusion of Patel et al. (2014) is an *in vitro* study in mouse hippocampal slices with electrophysiological stimulation, in the presence of glucose compared with pyruvate or, in some instances, lactate (Ivanov et al., 2014). The prime measures were changes in NAD(P)H and tissue oxygen, other measures being FAD, intracellular pH and Ca^2+^, and ATP (Ivanov et al., 2014). Distinction of neuronal contributions was achieved using inhibitors. These authors concluded, “Our data do not support the hypothesis of a preferential utilization of astrocyte-released lactate by neurons during network activation in slices–instead we show that during such activity glucose is an effective energy substrate for both neurons and astrocytes” (Ivanov et al., 2014).

Utilization of glucose but not lactate has been reported to correlate with N-methyl-D-aspartate (NMDA)-induced neurotransmission in mouse cerebellar neurons *in vitro* (Bak et al., 2009). These authors have suggested, “the role of extracellular lactate may be to support basal metabolism in neurons rather than neurotransmission activity *per se* mandating a revision of how we perceive the role of lactate in cerebral energy metabolism” (Bak et al., 2009).

Whether the above findings *in vivo* in rats, and *in vitro* in mouse brain slices and mouse neurons are relevant to human TBI is as yet unknown.

While it is still debated (Dienel, 2014) whether or not lactate is a “preferred fuel” (sparing glucose) after TBI or whether brain energetics are improved as a result of intravenous lactate, it is evident from ^13^C labeling studies that animal and human brain (injured and non-injured) can utilize lactate via the TCA cycle, judged by production of the appropriate ^13^C labeling patterns in glutamate and/or glutamine (Tyson et al., 2003; Gallagher et al., 2009; Boumezbeur et al., 2010). At face value, the idea that intravenous administration of exogenous lactate may be beneficial for TBI patients (Ichai et al., 2009, 2013; Bouzat et al., 2014) seems to conflict with the association between high (endogenous) brain extracellular lactate and poor outcome (Timofeev et al., 2011a). We have previously suggested that where build-up of (endogenous) lactate occurs, this may be because the neurons are too damaged to utilize it, which might explain the association between high endogenous extracellular lactate and poor outcome (Carpenter et al., 2014). A possible rationalization of the apparently beneficial effect of lactate infusion (Ichai et al., 2009, 2013; Bouzat et al., 2014) vs. the unfavorable association of high endogenous lactate (Timofeev et al., 2011a) might be that if PbtO_2_ is adequate, and if mitochondria are functioning, then brain cells can use lactate as a feedstock, via conversion to pyruvate and then acetate, for the TCA cycle and ultimately ATP production.

Whether other “alternative fuels” (apart from lactate) might be relevant in treating TBI is unknown. It has been suggested that sodium pyruvate might be preferable to sodium lactate, particularly as pyruvate would bypass the redox step from lactate to pyruvate (Dienel, 2014). However, pyruvate in solution is known to self-react, forming various products including a dimer (parapyruvate) that can, at least under some circumstances, inhibit alpha-ketoglutarate dehydrogenase, an enzyme in the TCA cycle (Margolis and Coxon, 1986). An alternative form of pyruvate that has been suggested is ethyl pyruvate which is reportedly more stable in solution and has anti-inflammatory effects distinct from sodium pyruvate (Fink, 2007).

## Conclusions and future prospects

Understanding of the roles of glycolysis and lactate in the human brain are continuing to evolve. From the original concept of lactate as merely a waste product, with adverse associations in injured brain, it is becoming increasingly recognized as a potential source of energy for the brain. Having the right balance between glycolysis (and PPP) and mitochondrial metabolism is vital. Controlling this balance is becoming recognized as a therapeutic target in treating TBI patients during neurocritical care. For interpreting the levels of brain extracellular lactate during multimodality monitoring it is important to also take into account the other parameters, including brain extracellular pyruvate and glucose levels, as well as circulating levels of glucose and lactate in blood. Infusion and perfusion studies, and labeling with ^13^C with detection by *ex vivo* NMR or *in vivo* MRS, have potential to shed further light on cerebral energy metabolism in human brain and may help suggest strategies for improving TBI treatment protocols for better clinical outcomes.

### Conflict of interest statement

Dr Peter J. Hutchinson is a Director of Technicam. The authors declare that the research was conducted in the absence of any commercial or financial relationships that could be construed as a potential conflict of interest.

## References

[B1] Alessandri B. Schwandt E. Kamada Y. Nagata M. Heimann A. Kempski O. (2012). The neuroprotective effect of lactate is not due to improved glutamate uptake after controlled cortical impact in rats. J. Neurotrauma 29, 2181–2191. 10.1089/neu.2011.2067 22888957

[B2] Bak L. K. Walls A. B. Schousboe A. Ring A. Sonnewald U. Waagepetersen H. S. (2009). Neuronal glucose but not lactate utilization is positively correlated with NMDA-induced neurotransmission and fluctuations in cytosolic Ca2+ levels. J. Neurochem. 109 (Suppl. 1), 87–93. 10.1111/j.1471-4159.2009.05943.x 19393013

[B3] Bartnik B. L. Lee S. M. Hovda D. A. Sutton R. L. (2007). The fate of glucose during the period of decreased metabolism after fluid percussion injury: a 13C NMR study. J. Neurotrauma 24, 1079–1092. 10.1089/neu.2006.0210 17610349

[B4] Bartnik B. L. Sutton R. L. Fukushima M. Harris N. G. Hovda D. A. Lee S. M. (2005). Upregulation of pentose phosphate pathway and preservation of tricarboxylic acid cycle flux after experimental brain injury. J. Neurotrauma 22, 1052–1065. 10.1089/neu.2005.22.1052 16238483

[B5] Befroy D. E. Rothman D. L. Petersen K. F. Shulman G. I. (2012). (31)P-magnetization transfer magnetic resonance spectroscopy measurements of *in vivo* metabolism. Diabetes 61, 2669–2678. 10.2337/db12-0558 23093656 PMC3478545

[B6] Bensinger S. J. Christofk H. R. (2012). New aspects of the Warburg effect in cancer cell biology. Semin. Cell Dev. Biol. 23, 352–361. 10.1016/j.semcdb.2012.02.003 22406683

[B7] Ben-Yoseph O. Boxer P. A. Ross B. D. (1996). Assessment of the role of the glutathione and pentose phosphate pathways in the protection of primary cerebrocortical cultures from oxidative stress. J. Neurochem. 66, 2329–2337. 10.1046/j.1471-4159.1996.66062329.x 8632155

[B8] Beutler E. Yeh M. Fairbanks V. F. (1962). The normal human female as a mosaic of X-chromosome activity: studies using the gene for C-6-PD-deficiency as a marker. Proc. Natl. Acad. Sci. U.S.A. 48, 9–16. 10.1073/pnas.48.1.9 13868717 PMC285481

[B9] Bittar P. G. Charnay Y. Pellerin L. Bouras C. Magistretti P. J. (1996). Selective distribution of lactate dehydrogenase isoenzymes in neurons and astrocytes of human brain. J. Cereb. Blood Flow Metab. 16, 1079–1089. 10.1097/00004647-199611000-00001 8898679

[B10] Boumezbeur F. Petersen K. F. Cline G. W. Mason G. F. Behar K. L. Shulman G. I.. (2010). The contribution of blood lactate to brain energy metabolism in humans measured by dynamic 13C nuclear magnetic resonance spectroscopy. J. Neurosci. 30, 13983–13991. 10.1523/JNEUROSCI.2040-10.2010 20962220 PMC2996729

[B11] Bouzat P. Sala N. Suys T. Zerlauth J. B. Marques-Vidal P. Feihl F.. (2014). Cerebral metabolic effects of exogenous lactate supplementation on the injured human brain. Intensive Care Med. 40, 412–421. 10.1007/s00134-013-3203-6 24477453

[B12] Brekke E. M. Morken T. S. Wideroe M. Haberg A. K. Brubakk A. M. Sonnewald U. (2014). The pentose phosphate pathway and pyruvate carboxylation after neonatal hypoxic-ischemic brain injury. J. Cereb. Blood Flow Metab. 34, 724–734. 10.1038/jcbfm.2014.8 24496178 PMC3982102

[B13] Cappellini M. D. Fiorelli G. (2008). Glucose-6-phosphate dehydrogenase deficiency. Lancet 371, 64–74. 10.1016/S0140-6736(08)60073-2 18177777

[B14] Carpenter K. L. Jalloh I. Gallagher C. N. Grice P. Howe D. J. Mason A.. (2014). (13)C-labelled microdialysis studies of cerebral metabolism in TBI patients. Eur. J. Pharm. Sci. 57, 87–97. 10.1016/j.ejps.2013.12.012 24361470 PMC4013834

[B15] Chesnut R. M. Marshall L. F. Klauber M. R. Blunt B. A. Baldwin N. Eisenberg H. M.. (1993). The role of secondary brain injury in determining outcome from severe head injury. J. Trauma 34, 216–222. 10.1097/00005373-199302000-00006 8459458

[B16] Chih C. P. Roberts E. L. Jr. (2003). Energy substrates for neurons during neural activity: a critical review of the astrocyte-neuron lactate shuttle hypothesis. J. Cereb. Blood Flow Metab. 23, 1263–1281. 10.1097/01.WCB.0000081369.51727.6F 14600433

[B17] Cruz F. Cerdan S. (1999). Quantitative 13C NMR studies of metabolic compartmentation in the adult mammalian brain. NMR Biomed. 12, 451–462. 10654292 10.1002/(sici)1099-1492(199911)12:7<451::aid-nbm571>3.0.co;2-e

[B18] De Bock K. Georgiadou M. Schoors S. Kuchnio A. Wong B. W. Cantelmo A. R.. (2013). Role of PFKFB3-driven glycolysis in vessel sprouting. Cell 154, 651–663. 10.1016/j.cell.2013.06.037 23911327

[B19] Dienel G. A. (2014). Lactate shuttling and lactate use as fuel after traumatic brain injury: metabolic considerations. J. Cereb. Blood Flow Metab. 34, 1736–1748. 10.1038/jcbfm.2014.153 25204393 PMC4269761

[B20] Domanska-Janik K. (1988). Hexose monophosphate pathway activity in normal and hypoxic rat brain. Resuscitation 16, 79–90. 10.1016/0300-9572(88)90073-1 2839885

[B21] Dusick J. R. Glenn T. C. Lee W. N. Vespa P. M. Kelly D. F. Lee S. M.. (2007). Increased pentose phosphate pathway flux after clinical traumatic brain injury: a [1,2-13C2]glucose labeling study in humans. J. Cereb. Blood Flow Metab. 27, 1593–1602. 10.1038/sj.jcbfm.9600458 17293841

[B22] Fink M. P. (2007). Ethyl pyruvate: a novel anti-inflammatory agent. J. Intern. Med. 261, 349–362. 10.1111/j.1365-2796.2007.01789.x 17391109

[B23] Gallagher C. N. Carpenter K. L. Grice P. Howe D. J. Mason A. Timofeev I. (2009). The human brain utilizes lactate via the tricarboxylic acid cycle: a 13C-labelled microdialysis and high-resolution nuclear magnetic resonance study. Brain 132(Pt 10), 2839–2849 10.1093/brain/awp202 19700417

[B24] Galow L. V. Schneider J. Lewen A. Ta T. T. Papageorgiou I. E. Kann O. (2014). Energy substrates that fuel fast neuronal network oscillations. Front. Neurosci. 8:398. 10.3389/fnins.2014.00398 25538552 PMC4256998

[B25] Garnett M. R. Corkill R. G. Blamire A. M. Rajagopalan B. Manners D. N. Young J. D.. (2001). Altered cellular metabolism following traumatic brain injury: a magnetic resonance spectroscopy study. J. Neurotrauma 18, 231–240. 10.1089/08977150151070838 11284544

[B26] Gendrel A. V. Heard E. (2011). Fifty years of X-inactivation research. Development 138, 5049–5055. 10.1242/dev.068320 22069183

[B27] Harper P. S. (2011). Mary Lyon and the hypothesis of random X chromosome inactivation. Hum. Genet. 130, 169–174. 10.1007/s00439-011-1013-x 21643983

[B28] Holloway R. Zhou Z. Harvey H. B. Levasseur J. E. Rice A. C. Sun D.. (2007). Effect of lactate therapy upon cognitive deficits after traumatic brain injury in the rat. Acta Neurochir. (Wien) 149, 919–927; discussion 927. 10.1007/s00701-007-1241-y 17660938

[B29] Hutchinson P. J. O'Connell M. T. Seal A. Nortje J. Timofeev I. Al-Rawi P. G.. (2009). A combined microdialysis and FDG-PET study of glucose metabolism in head injury. Acta Neurochir. (Wien) 151, 51–61; discussion 61. 10.1007/s00701-008-0169-1 19099177

[B30] Ichai C. Armando G. Orban J. C. Berthier F. Rami L. Samat-Long C.. (2009). Sodium lactate versus mannitol in the treatment of intracranial hypertensive episodes in severe traumatic brain-injured patients. Intensive Care Med. 35, 471–479. 10.1007/s00134-008-1283-5 18807008

[B31] Ichai C. Payen J. F. Orban J. C. Quintard H. Roth H. Legrand R.. (2013). Half-molar sodium lactate infusion to prevent intracranial hypertensive episodes in severe traumatic brain injured patients: a randomized controlled trial. Intensive Care Med. 39, 1413–1422. 10.1007/s00134-013-2978-9 23749153

[B32] Ide K. Schmalbruch I. K. Quistorff B. Horn A. Secher N. H. (2000). Lactate, glucose and O2 uptake in human brain during recovery from maximal exercise. J. Physiol. 522(Pt 1), 159–164. 10.1111/j.1469-7793.2000.t01-2-00159.xm 10618160 PMC2269743

[B33] Ivanov A. I. Malkov A. E. Waseem T. Mukhtarov M. Buldakova S. Gubkina O.. (2014). Glycolysis and oxidative phosphorylation in neurons and astrocytes during network activity in hippocampal slices. J. Cereb. Blood Flow Metab. 34, 397–407. 10.1038/jcbfm.2013.222 24326389 PMC3948126

[B34] Jalloh I. Carpenter K. L. Grice P. Howe D. J. Mason A. Gallagher C. N.. (2015). Glycolysis and the pentose phosphate pathway after human traumatic brain injury: microdialysis studies using 1,2-(13)C2 glucose. J. Cereb. Blood Flow Metab. 35, 111–120. 10.1038/jcbfm.2014.177 25335801 PMC4294402

[B35] Jalloh I. Carpenter K. L. Helmy A. Carpenter T. A. Menon D. K. Hutchinson P. J. (2014). Glucose metabolism following human traumatic brain injury: methods of assessment and pathophysiological findings. Metab. Brain Dis. 10.1007/s11011-014-9628-y[Epub ahead of print]. 25413449 PMC4555200

[B36] Jalloh I. Helmy A. Shannon R. J. Gallagher C. N. Menon D. K. Carpenter K. L.. (2013). Lactate uptake by the injured human brain: evidence from an arteriovenous gradient and cerebral microdialysis study. J. Neurotrauma 30, 2031–2037. 10.1089/neu.2013.2947 23968221 PMC3868386

[B37] John S. Weiss J. N. Ribalet B. (2011). Subcellular localization of hexokinases I and II directs the metabolic fate of glucose. PLoS ONE 6:e17674. 10.1371/journal.pone.0017674 21408025 PMC3052386

[B38] Katayama Y. Becker D. P. Tamura T. Hovda D. A. (1990). Massive increases in extracellular potassium and the indiscriminate release of glutamate following concussive brain injury. J. Neurosurg. 73, 889–900. 10.3171/jns.1990.73.6.0889 1977896

[B39] Kawamata T. Katayama Y. Hovda D. A. Yoshino A. Becker D. P. (1992). Administration of excitatory amino acid antagonists via microdialysis attenuates the increase in glucose utilization seen following concussive brain injury. J. Cereb. Blood Flow Metab. 12, 12–24. 10.1038/jcbfm.1992.3 1345756

[B40] Kramer A. H. Roberts D. J. Zygun D. A. (2012). Optimal glycemic control in neurocritical care patients: a systematic review and meta-analysis. Crit. Care 16, R203. 10.1186/cc11812 23082798 PMC3682305

[B41] Lebon V. Petersen K. F. Cline G. W. Shen J. Mason G. F. Dufour S.. (2002). Astroglial contribution to brain energy metabolism in humans revealed by 13C nuclear magnetic resonance spectroscopy: elucidation of the dominant pathway for neurotransmitter glutamate repletion and measurement of astrocytic oxidative metabolism. J. Neurosci. 22, 1523–1531. 11880482 10.1523/JNEUROSCI.22-05-01523.2002 PMC2995528

[B42] Lewen A. Fujimura M. Sugawara T. Matz P. Copin J. C. Chan P. H. (2001). Oxidative stress-dependent release of mitochondrial cytochrome c after traumatic brain injury. J. Cereb. Blood Flow Metab. 21, 914–920. 10.1097/00004647-200108000-00003 11487726

[B43] Lyon M. F. (1961). Gene action in the X-chromosome of the mouse (*Mus musculus* L.). Nature 190, 372–373. 10.1038/190372a0 13764598

[B44] Maddock R. J. Buonocore M. H. (2012). MR spectroscopic studies of the brain in psychiatric disorders. Curr. Top. Behav. Neurosci. 11, 199–251. 10.1007/7854_2011_197 22294088

[B45] Margolis S. A. Coxon B. (1986). Identification and quantitation of the impurities in sodium pyruvate. Anal. Chem. 58, 2504–2510 10.1021/ac00125a033

[B46] Marino S. Zei E. Battaglini M. Vittori C. Buscalferri A. Bramanti P.. (2007). Acute metabolic brain changes following traumatic brain injury and their relevance to clinical severity and outcome. J. Neurol. Neurosurg. Psychiatry 78, 501–507. 10.1136/jnnp.2006.099796 17088335 PMC2117835

[B47] Neely J. R. Denton R. M. England P. J. Randle P. J. (1972). The effects of increased heart work on the tricarboxylate cycle and its interactions with glycolysis in the perfused rat heart. Biochem. J. 128, 147–159. 5085551 10.1042/bj1280147 PMC1173579

[B48] Newcombe V. F. Williams G. B. Outtrim J. G. Chatfield D. Gulia Abate M. Geeraerts T.. (2013). Microstructural basis of contusion expansion in traumatic brain injury: insights from diffusion tensor imaging. J. Cereb. Blood Flow Metab. 33, 855–862. 10.1038/jcbfm.2013.11 23423189 PMC3677102

[B49] Notaro R. Afolayan A. Luzzatto L. (2000). Human mutations in glucose 6-phosphate dehydrogenase reflect evolutionary history. FASEB J. 14, 485–494. 10698963 10.1096/fasebj.14.3.485

[B50] Overgaard M. Rasmussen P. Bohm A. M. Seifert T. Brassard P. Zaar M.. (2012). Hypoxia and exercise provoke both lactate release and lactate oxidation by the human brain. FASEB J. 26, 3012–3020. 10.1096/fj.11-191999 22441982

[B51] Pandolfi P. P. Sonati F. Rivi R. Mason P. Grosveld F. Luzzatto L. (1995). Targeted disruption of the housekeeping gene encoding glucose 6-phosphate dehydrogenase (G6PD): G6PD is dispensable for pentose synthesis but essential for defense against oxidative stress. EMBO J. 14, 5209–5215. 7489710 10.1002/j.1460-2075.1995.tb00205.x PMC394630

[B52] Patel A. B. Lai J. C. Chowdhury G. M. Hyder F. Rothman D. L. Shulman R. G.. (2014). Direct evidence for activity-dependent glucose phosphorylation in neurons with implications for the astrocyte-to-neuron lactate shuttle. Proc. Natl. Acad. Sci. U.S.A. 111, 5385–5390. 10.1073/pnas.1403576111 24706914 PMC3986127

[B53] Pellerin L. Magistretti P. J. (1994). Glutamate uptake into astrocytes stimulates aerobic glycolysis: a mechanism coupling neuronal activity to glucose utilization. Proc. Natl. Acad. Sci. U.S.A. 91, 10625–10629. 10.1073/pnas.91.22.10625 7938003 PMC45074

[B54] Pellerin L. Magistretti P. J. (2012). Sweet sixteen for ANLS. J. Cereb. Blood Flow Metab. 32, 1152–1166. 10.1038/jcbfm.2011.149 22027938 PMC3390819

[B55] Rice A. C. Zsoldos R. Chen T. Wilson M. S. Alessandri B. Hamm R. J.. (2002). Lactate administration attenuates cognitive deficits following traumatic brain injury. Brain Res. 928, 156–159. 10.1016/S0006-8993(01)03299-1 11844482

[B56] Riganti C. Gazzano E. Polimeni M. Aldieri E. Ghigo D. (2012). The pentose phosphate pathway: an antioxidant defense and a crossroad in tumor cell fate. Free Radic. Biol. Med. 53, 421–436. 10.1016/j.freeradbiomed.2012.05.006 22580150

[B57] Rodriguez-Rodriguez P. Fernandez E. Bolanos J. P. (2013). Underestimation of the pentose-phosphate pathway in intact primary neurons as revealed by metabolic flux analysis. J. Cereb. Blood Flow Metab. 33, 1843–1845. 10.1038/jcbfm.2013.168 24064491 PMC3851909

[B58] Roozenbeek B. Maas A. I. Menon D. K. (2013). Changing patterns in the epidemiology of traumatic brain injury. Nat. Rev. Neurol. 9, 231–236. 10.1038/nrneurol.2013.22 23443846

[B59] Rothman D. L. De Feyter H. M. de Graaf R. A. Mason G. F. Behar K. L. (2011). 13C MRS studies of neuroenergetics and neurotransmitter cycling in humans. NMR Biomed. 24, 943–957. 10.1002/nbm.1772 21882281 PMC3651027

[B60] Sala N. Suys T. Zerlauth J. B. Bouzat P. Messerer M. Bloch J.. (2013). Cerebral extracellular lactate increase is predominantly nonischemic in patients with severe traumatic brain injury. J. Cereb. Blood Flow Metab. 33, 1815–1822. 10.1038/jcbfm.2013.142 23963367 PMC3824185

[B61] Samaras N. Samaras D. Frangos E. Forster A. Philippe J. (2013). A review of age-related dehydroepiandrosterone decline and its association with well-known geriatric syndromes: is treatment beneficial? Rejuvenation Res. 16, 285–294. 10.1089/rej.2013.1425 23647054 PMC3746247

[B62] Schumacher M. Weill-Engerer S. Liere P. Robert F. Franklin R. J. Garcia-Segura L. M.. (2003). Steroid hormones and neurosteroids in normal and pathological aging of the nervous system. Prog. Neurobiol. 71, 3–29. 10.1016/j.pneurobio.2003.09.004 14611864

[B63] Schurr A. (2006). Lactate: the ultimate cerebral oxidative energy substrate? J. Cereb. Blood Flow Metab. 26, 142–152. 10.1038/sj.jcbfm.9600174 15973352

[B64] Shannon R. J. Carpenter K. L. Guilfoyle M. R. Helmy A. Hutchinson P. J. (2013). Cerebral microdialysis in clinical studies of drugs: pharmacokinetic applications. J. Pharmacokinet. Pharmacodyn. 40, 343–358. 10.1007/s10928-013-9306-4 23468415 PMC3663257

[B65] Sonnewald U. (2014). Glutamate synthesis has to be matched by its degradation - where do all the carbons go? J. Neurochem. 131, 399–406. 10.1111/jnc.12812 24989463

[B66] Stocchetti N. Furlan A. Volta F. (1996). Hypoxemia and arterial hypotension at the accident scene in head injury. J. Trauma 40, 764–767. 10.1097/00005373-199605000-00014 8614077

[B67] Suzuki A. Stern S. A. Bozdagi O. Huntley G. W. Walker R. H. Magistretti P. J.. (2011). Astrocyte-neuron lactate transport is required for long-term memory formation. Cell 144, 810–823. 10.1016/j.cell.2011.02.018 21376239 PMC3073831

[B68] TeSlaa T. Teitell M. A. (2014). Techniques to monitor glycolysis. Methods Enzymol. 542, 91–114. 10.1016/B978-0-12-416618-9.00005-4 24862262 PMC4276425

[B69] Timofeev I. Carpenter K. L. Nortje J. Al-Rawi P. G. O'Connell M. T. Czosnyka M. (2011a). Cerebral extracellular chemistry and outcome following traumatic brain injury: a microdialysis study of 223 patients. Brain 134(Pt 2), 484–494 10.1093/brain/awq353 21247930

[B70] Timofeev I. Czosnyka M. Carpenter K. L. Nortje J. Kirkpatrick P. J. Al-Rawi P. G.. (2011b). Interaction between brain chemistry and physiology after traumatic brain injury: impact of autoregulation and microdialysis catheter location. J. Neurotrauma 28, 849–860. 10.1089/neu.2010.1656 21488707 PMC3113421

[B71] Timofeev I. Nortje J. Al-Rawi P. G. Hutchinson P. J. Gupta A. K. (2013). Extracellular brain pH with or without hypoxia is a marker of profound metabolic derangement and increased mortality after traumatic brain injury. J. Cereb. Blood Flow Metab. 33, 422–427. 10.1038/jcbfm.2012.186 23232949 PMC3587815

[B72] Tyson R. L. Gallagher C. Sutherland G. R. (2003). 13C-Labeled substrates and the cerebral metabolic compartmentalization of acetate and lactate. Brain Res. 992, 43–52. 10.1016/j.brainres.2003.08.027 14604771

[B73] Unterberg A. W. Stover J. Kress B. Kiening K. L. (2004). Edema and brain trauma. Neuroscience 129, 1021–1029. 10.1016/j.neuroscience.2004.06.046 15561417

[B74] van Hall G. Stromstad M. Rasmussen P. Jans O. Zaar M. Gam C.. (2009). Blood lactate is an important energy source for the human brain. J. Cereb. Blood Flow Metab. 29, 1121–1129. 10.1038/jcbfm.2009.35 19337275

[B75] Vespa P. Bergsneider M. Hattori N. Wu H. M. Huang S. C. Martin N. A.. (2005). Metabolic crisis without brain ischemia is common after traumatic brain injury: a combined microdialysis and positron emission tomography study. J. Cereb. Blood Flow Metab. 25, 763–774. 10.1038/sj.jcbfm.9600073 15716852 PMC4347944

[B76] Warburg O. (1956). On the origin of cancer cells. Science 123, 309–314. 10.1126/science.123.3191.309 13298683

[B77] Xiong Y. Gu Q. Peterson P. L. Muizelaar J. P. Lee C. P. (1997). Mitochondrial dysfunction and calcium perturbation induced by traumatic brain injury. J. Neurotrauma 14, 23–34. 10.1089/neu.1997.14.23 9048308

